# Detection of Endoparasites in Non-Native Raccoons from Central Italy

**DOI:** 10.3390/vetsci10020171

**Published:** 2023-02-20

**Authors:** Andrea Lombardo, Marco Diano, Giuseppina Brocherel, Lucia Palmerini, Serena Giovannini, Ziad Mezher, Manuela Iurescia, Tamara Cerci, Andrea Caprioli, Claudia Eleni, Caterina Raso, Alessia Mariacher, Irene Del Lesto, Nadia Cappai, Luca Mattioli, Claudio De Liberato, Gianluca Fichi

**Affiliations:** 1 Istituto Zooprofilattico Sperimentale del Lazio e Della Toscana “M. Aleandri”, 52100 Arezzo, Italy; 2 Istituto Zooprofilattico Sperimentale del Lazio e Della Toscana “M. Aleandri”, 53100 Siena, Italy; 3 Istituto Zooprofilattico Sperimentale del Lazio e Della Toscana “M. Aleandri”, 00178 Roma, Italy; 4 Istituto Zooprofilattico Sperimentale del Lazio e Della Toscana “M. Aleandri”, 02100 Rieti, Italy; 5 Istituto Zooprofilattico Sperimentale del Lazio e Della Toscana “M. Aleandri”, 58100 Grosseto, Italy; 6 Parco Nazionale Foreste Casentinesi, Monte Falterona e Campigna, 52015 Pratovecchio, Italy; 7 Regione Toscana, Presidio Territoriale del Settore Faunistico Venatorio ed Ittico, 52100 Arezzo, Italy

**Keywords:** raccoon, *Baylisascaris procyonis*, zoonosis, endoparasites, *Cryptosporidium*, *Pearsonema*, Ancylostomatidae, Italy

## Abstract

**Simple Summary:**

The raccoon (*Procyon lotor*) is a carnivore native to North and Central America, gradually introduced into Asia and Europe, including Italy. It is an important carrier of multiple endoparasites, some of them being zoonotic. The aim of this study was to investigate the endoparasites of the non-native raccoon population of Central Italy. The results showed the presence of a number of different Protozoa and Nematodes. Among them, we identified *Baylisascaris procyonis*, an emerging helminthic zoonotic agent of serious concern for public and animal health, given the possibility of its transmission to paratenic hosts, including humans and pets, at times with severe clinical consequences. The role of the raccoon as a multi-parasite carrier highlights the importance of the eradication/control of this alien species and the need to implement related disease surveillance programs.

**Abstract:**

The raccoon (*Procyon lotor*) is a carnivore native to North and Central America, gradually introduced into Asia and Europe, including Italy. It is an important carrier of multiple endoparasites, both Protozoa and Helminths, some of them being zoonotic. The aim of this study was to investigate the endoparasites of the non-native raccoon population of Central Italy. Sixty-two raccoons were collected by local competent authorities (sixty trapped and euthanized, two found dead) and subjected to necroscopic examination. Carcasses underwent a broad parasitological investigation, including coprological techniques (macroscopic examination of the gastrointestinal tract, lungs, trachea, and heart, Flotac^®^, Baermann test, and immunofluorescence for *Giardia duodenalis* and *Cryptosporidium* spp.), research on respiratory/urinary capillariosis and artificial digestion for *Trichinella* spp. larvae, and a histopathological examination of the ileum. Ascarid parasites were further identified at the species level using a next-generation sequencing-based amplicon sequencing approach. The results showed the presence of different Protozoa and Nematodes: *Baylisascaris procyonis* (26/62; 41.9%), *Pearsonema* sp. (6/62; 9.6%), Capillariidae (6/62; 9.6%), *Eimeria* sp. (2/62; 3.2%), *Cryptosporidium* sp. (2/62; 3.2%), and Ancylostomatidae (2/62; 3.2%). *B. procyonis* is an emerging helminthic zoonotic agent considered a serious concern for public and animal health, given the possibility of its transmission to paratenic hosts, including humans and pets. The demonstrated role of the raccoon as a multi-parasite carrier should be an incentive to continuing the eradication/control of this alien species, and supports the need to implement related disease surveillance programs.

## 1. Introduction

The raccoon (*Procyon lotor*) is a common mesopredator native to most of North and Central America that has been gradually introduced into parts of Europe and Asia [1]. The ecological impacts of non-native raccoons encompass predation on native fauna, competition with other autochthonous carnivores, and the introduction and spreading of new pathogens [2,3]. The European Union lists the raccoon among the invasive species of European concern (EU Regulation 1143/2014). Until recently, in Italy, the raccoon was present as two separate populations: the first was reported in 2004 along the southern part of the Adda river (Northern Italy), and the second was more recently (2013) reported in the Province of Arezzo (Tuscany) within the Casentino Valley (Central Italy). Since 2017, an official eradication plan has been implemented in the country following the Italian Regulation “Decreto legislativo 230/2017” [4], leading to the eradication of the Northern Italy population [5]. In Tuscany, the eradication plan is currently ongoing, accompanied by a sanitary investigation of the carcasses, in the context of a research program funded by the Italian Ministry of Health (Grant Number RC IZSLT 10/2021).

The raccoon can play a critical role as a host to many endoparasites and is an environmental shedder of zoonotic agents, such as the ascarid *Baylisascaris procyonis*, cause of *Baylisascaris* larva migrans, an emerging helminthic zoonosis [2,6,7]. Its prevalence in free-ranging raccoons can be very high, especially in North America, exceeding 70% [2]. From the 1830s, following the spreading of the raccoons through the Old World, this parasite progressively increased its geographical range and, at present, it has been reported in non-native raccoon populations from many European countries, including Denmark [8], Austria [9], Poland [10] and Germany [11]. More recently, *B. procyonis* was reported in the Central Italy raccoon population by our research group [12]. As for other helminthic infections, numerous species other than *B. procyonis* were reported worldwide in raccoons, such as *Strongyloides procyonis, Aonchotheca putorii*, *Ancylostoma* sp., *Uncinaria* sp., *Trichuris* sp., *Trichinella pseudospiralis* [13], *Trichinella* T9 [14], *Polymorphus minutus* [15], *Mesocestoides* sp. [10], *Pharingostomoides* sp. [16], and *Pearsonema* sp. [17]. With regard to both Italian raccoon populations, a previous study by Romeo et al. (2021) focused on gastrointestinal helminthic infections and highlighted the presence of *S. procyonis, A. putorii*, Ancylostomatidae, and Trichuridae [3]. Moreover, several protozoal infections have been described in the raccoon worldwide, such as *Cryptosporidium parvum* [18], *Giardia duodenalis* [19]*,* and *Eimeria* spp. [20]. The aim of this work was to investigate the endoparasites of the non-native raccoon population of Central Italy, with a main focus on zoonotic ones.

## 2. Materials and Methods

Between August 2020 and September 2022, 62 free-ranging raccoon carcasses (31 males and 31 females), 55 adults (88.7%) and 7 juveniles (11.3%), with an average bodyweight of 4.42 ± 1.72 kg (Table 1), were submitted to the Istituto Zooprofilattico Sperimentale del Lazio e Toscana “M. Aleandri” laboratories for necropsy examination.

Sixty raccoons were captured in 32 different sites by cage traps, while two were found dead by the local authorities, showing lesions consistent with a road traffic accident and predation, respectively. The used traps were kept in place all year round and were checked daily for the presence of trapped animals. Non-target animals were immediately released, while raccoons were euthanized by trained personnel from the local police authority or by wildlife veterinarians. Trapping and euthanasia were performed in the context of a state-instituted eradication program, with the aim of eradicating this alien species. No animal was trapped and/or killed with the aim of providing samples for this study and no live animal was involved in laboratory investigation. Thus, no other special permits were required to legally euthanize raccoons. Euthanasia protocols were designed following high animal welfare standards; according to Reg. 2010/63/EU Annex IV guidelines for “other carnivores”, animals were euthanized either by shooting with a free bullet or by anesthetic overdose. To induce a state of deep anesthesia, we used a mixture of 10 mg/kg body weight of ketamine (Lobotor^®^, Acme S.r.l., Cavriago (RE), Italy) and 0.1 mg/kg medetomidine (Domitor^®^, Vétoquinol Italia S.r.l., Bertinoro (FC), Italy). All drugs were mixed in the same syringe and injected in the quadriceps muscle with the animal in a containment cage equipped with a squeeze chute. If the level of anesthesia was not adequate, a second half dose was administered. When the level of anesthesia was adequate (palpebral reflex, response to tactile stimulus, and tongue relaxation absent), the cephalic vein was catheterized and fluids administration started. Euthanasia was performed with an intravenous injection of 0,5 mL/kg EV of Tanax^®^. Trapping, handling, and euthanasia procedures were performed in order to minimize pain, suffering, distress, or lasting harm.

At necropsy, gross examination was mainly focused on the detection of parasitic lesions and adult helminths. For this purpose, the heart, trachea, lungs and gastrointestinal tract were collected from each animal. Respiratory and cardiovascular systems were processed as described by Lemming et al. in 2020 [21]. Briefly, the cardiac ventricles and the major thoracic blood vessels (mainly pulmonary artery and aorta) were opened using scissors and examined both macroscopically and microscopically for adult worms using a stereomicroscope at 80–100× magnification. The blood vessels, trachea, and bronchial tree were dissected longitudinally using scissors or scalpels and inspected macroscopically for adult worms. Additionally, several parallel transversal incisions were made throughout the whole lungs using scalpels approximately 1–2 cm apart; the lung cut surfaces were inspected for parasites. Subsequently, the heart, blood vessels, trachea and lungs were flushed with approximately 500 mL tap water into 500 mL V-shaped recipients. The fluid was left to sediment for about 20 min. After discarding the supernatant, the sediment was transferred to microscopic slides for examination using a light microscope (100–400× magnification). The gastrointestinal tract was opened longitudinally with scissors and the content was macroscopically examined for the presence of parasites. The intestinal wall was further processed for adult-stage Cestoda parasites, by means of the sedimentation and counting technique (SCT), as described by Eckert in 2003 [22]. Then, the intestine was cut in approximately 20 cm segments. Samples of ileum and rectal content were preserved for histological and copromicroscopic techniques, as described below. The remaining segments of the intestine were transferred in glass recipients with 1 l of saline solution. After shaking, the intestinal wall was squeezed and discarded. The fluid containing intestinal material was sedimented several times for about 15 min, discarding the supernatant, until the sediment looked sufficiently clear. The sediment was examined in small portions of 5 mL, in sterile Petri plates using a stereomicroscope (100× magnification). Recovered parasites were preserved in 70% ethanol and subsequently submitted to morphologic and morphometric identification. In the case of ascarid parasites, fragments (approximately 1 cm long) of adult parasites (one specimen for each infected raccoon) were submitted for molecular identification using a next-generation sequencing (NGS)-based amplicon sequencing approach, as described by Lombardo et al. in 2022 [12]. Moreover, from each raccoon, samples of rectal content, ileum, skeletal muscles (diaphragm and tibialis muscles), urine, and nasal (choanae) lavage fluid were also collected. Feces from the rectum were submitted to a broad parasitological investigation encompassing Flotac^®^, Baermann test, immunofluorescence (IF) for *Giardia* cysts and *Cryptosporidium* oocysts (Merifluor^®^ *Cryptosporidium/Giardia* direct fluorescence assay), and quali-quantitative evaluation of larvae/eggs/cysts/oocysts. The Baermann test and Flotac^®^ were performed as described by Cringoli et al. in 2010 [23]. In order to detect early-stage infections caused by *B. procyonis* larvae penetrating the intestinal submucosa, samples of ileum were fixed in 10% neutral buffered formalin, embedded in paraffin wax, sectioned at 4 µm, and routinely stained with hematoxylin and eosin (HE) for histological examination. Diaphragm and tibialis muscles were collected (approximately 50 g for each raccoon) and examined for *Trichinella* spp. infection by artificial digestion, according to EU Reg. 1375/2015. Respiratory (*Eucoleus* spp.) and urinary (*Pearsonema* spp.) capillariid parasites were investigated from nasal and urinary samples, respectively. Nasal cavities were first inspected macroscopically; flushing was performed using approximately 30 mL of sterile saline solution in a syringe. Choanae were pressure-washed and the flushing saline solution was collected from nostrils in sterile tubes. Approximately 5 mL of urine was collected in sterile tubes by cystocentesis, using sterile syringes. The urinary bladder was also inspected macroscopically and microscopically, using a stereomicroscope (80–100× magnifications). Urine and nasal lavage fluids were analyzed for the presence of adult Capillariidae and centrifuged (0.941× *g* 5 min), and the supernatant was discarded. About a 0.1 mL droplet was suspended and floated in a NaCl saturated solution, and then microscopically examined [24].

In order to perform a spatial epidemiological analysis, the QGIS software was used. The coordinates of the trapping sites and of the traps where positive *B. procyonis* raccoons were captured were recorded. The number of raccoons that were captured with each trap, as well as the average raccoon home range, were considered in the analysis in order to identify possible areas with higher density of animals. Statistical analyses were carried out using R software [25]. The observed values were fitted into a contingency table in order to evaluate the possible relationships between the variables (sex, age, seasonality, and weight) and the presence of *B. procyonis*. We applied the Chi-square test of independence to the categorical variables and converted the quantitative variable (weight) into a categorical variable by separating the observations into intervals.

## 3. Results

### 3.1. Endoparasites Detection

The most prevalent detected parasite was the ascarid *B. procyonis* (26/62; 41.9%), followed by *Pearsonema* sp. (6/62; 9.6%), Capillariidae (6/62; 9.6%), *Eimeria* sp. (2/62; 3.2%), *Cryptosporidium* sp. (2/62; 3.2%), and Ancylostomatidae (2/62; 3.2%). Three trapped raccoons showed coinfections due to *B. procyonis* and *Eimeria* sp. (1/62; 1.6%), or *B. procyonis* and Ancylostomatidae (2/62; 3.2%). All raccoons tested negative for *G. duodenalis*, *Trichinella* spp. and cardio-pulmonary nematodes. For *Pearsonema* sp., only one female adult parasite was recovered from urine. For other Capillariidae and Ancylostomatidae, no larval or adult parasite was detected using the sedimentation techniques.

Quali-quantitative parasitological results from feces collected from the rectum and from urine sediments are summarized in Table 2. 

Regarding *B. procyonis*, 24/62 raccoons were positive (38.7%), both at macroscopic examination of the ileal lumen and at coproparasitology; one animal harbored just one female specimen (thus being negative at coproparasitology), while one showed a prepatent infection with histologically evident larvae in the ileal mucosa, without showing adults parasites or eggs. *B. procyonis* loads estimated by counting adult parasites in the ileal lumen are presented in Table 3. 

### 3.2. B. procyonis Identification

Morphological and morphometric features of all the ascarids detected were consistent with those of *B. procyonis*: length 9–16 cm, width 1–3 mm, white-yellowish to tan-colored, with a prominent dark alimentary tract. Male parasites showed typical features of the genus *Baylisascaris*, such as the presence of cervical alae with cuticular bars, dorsal and subventral labial papillae distinctly double, pericloacal area rugosa, uniform spicules, and discrete precloacal and postcloacal groups of papillae on the tail [12]. Molecular analyses of twenty-five adult parasites (one for each positive raccoon) confirmed the phenotypic identification, resulting in a 100% coverage and identity with a *B. procyonis*-deposited sequence (GenBank accession number: MZ092853), and our previously deposited sequence (GenBank accession number: OU974763.1) [12].

### 3.3. Pathology and Histopathology

Macroscopically, five raccoons showed intestinal obstruction due to a high parasite burden (>100 specimens, Table 3). These animals showed poor body conditions; large amounts of adult parasites were tangled up in the ileum lumen, preventing the normal transit of feces. The intestinal wall showed distention, proximal to the obstruction, and a lack of fecal material downstream. No other relevant gross lesions were observed.

**Figure 1:**
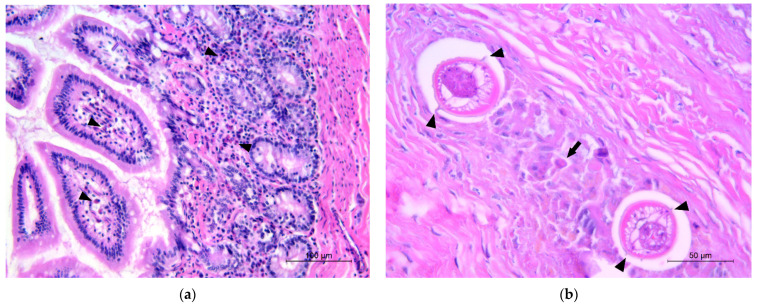
Histological sections of raccoon intestine. (**a**) Small eosinophilic aggregates in the lamina propria (black arrowheads); (**b**) larvae of *B. procyonis* in the ileal submucosa showing prominent lateral alae (black arrowhead) in the cross-section, associated with macrophages, eosinophils (black arrow), and cellular debris.

The histopathological examination of the ileum revealed eosinophilic enteritis, ranging from mild to severe, in all the animals in which adult *B. procyonis* was detected. The eosinophils were scattered or arranged in small aggregates and cords in the intestinal crypts, lamina propria, and submucosa (Figure 1a); in one case, eosinophilic infiltration was extended to the ileal *tunica muscularis* and mesenteric adipose tissue, and in three other cases, lymphocytes and plasma cells were also observed, indicating a chronicity of the inflammatory process. In the ileal submucosa of three animals, the histological examination showed the presence of granulomatous lesions, with larvae referable to *B. procyonis* as described by Kazacos in 2001 [2], surrounded by inflammatory exudate consisting of macrophages, eosinophils, lymphocytes, plasma cells, and cellular debris (Figure 1b, Figures S1 and S2). Two of these cases were related to the presence of adults in the intestinal lumen, while the other one did not show this correlation. No significant microscopic lesions were seen in the intestines of negative animals.

### 3.4. Spatial Distribution of B. procyonis Positive Raccoons

**Figure 2:**
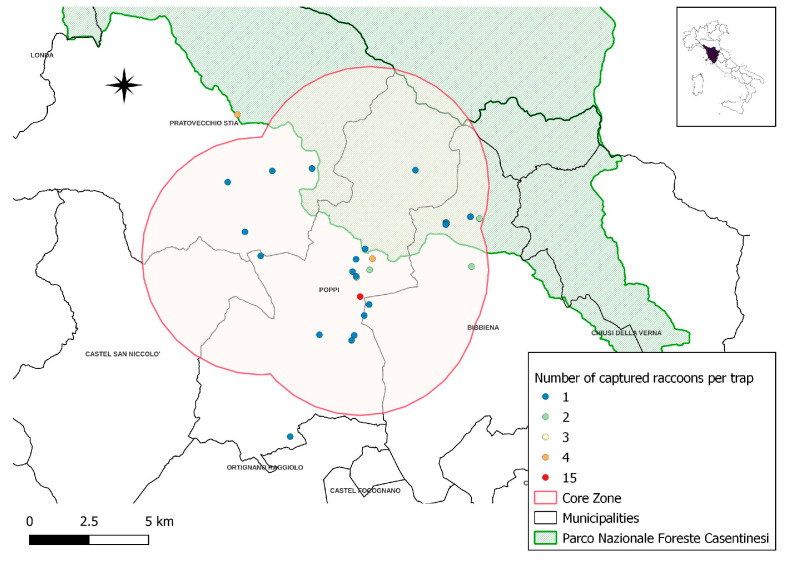
Raccoon-trapping sites (the proximity of some trapping sites results in the overlapping of dots in the picture). The red areas represent the raccoon “core zone”.

**Figure 3:**
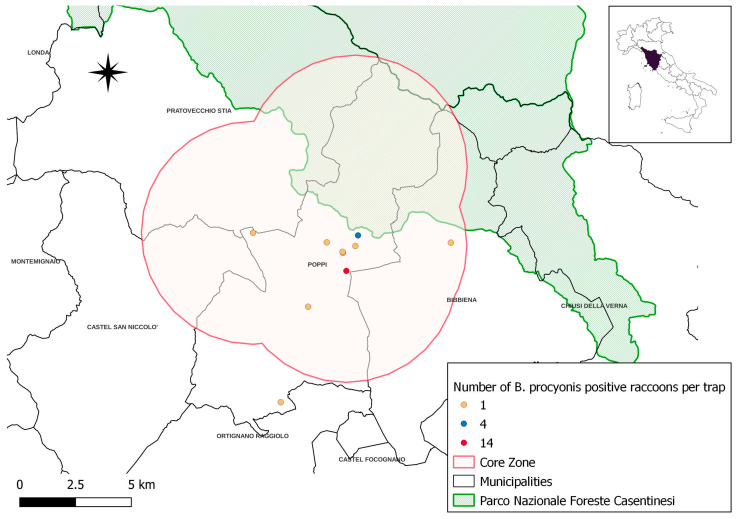
Sites of trapped *B. procyonis*-positive raccoons (the proximity of some trapping sites results in the overlapping of dots in the picture).

The trapping sites (*n* = 32) and the number of raccoons captured with each trap (*n* = 60) are represented in Figure 2 and Table 4 and Table S1. Based on these data and considering the average raccoon home range (5 km^2^) [26], we identified a core zone where the population seems to be mostly concentrated (55/60 trapped raccoons) and where most of the *B. procyonis*-positive animals were detected (Figure 3). This area has a surface of 160 km^2^ and encompasses parts of four different municipalities.

Almost 70% of *B. procyonis*-positive raccoons (18/26, 69.2%) were captured in just two trapping sites (Figure 3, red and blue dots on the map). Each of the remaining eight positive raccoons were captured within different trapping sites (Figure 3, yellow dots on the map). Chi-square tests did not show any significant relationship between the presence of *B. procyonis* and the considered variables (sex, age, seasonality, and weight) (Table 5). 

## 4. Discussion

In accordance with the previous literature, the results of this parasitological investigation confirm the role of the raccoon as a host for several species of endoparasites, both Protozoa [27] and Helminths [28,29,30,31]. Among protozoal infections, cryptosporidiosis of raccoons has been documented in several countries: Illinois and Virginia, USA [18,32,33], Luxembourg [34], Poland [35], Germany [36], Iran [37], and Japan [27]. Cryptosporidiosis is a disease of significant human health concern because of the possibility of its transmission through contaminated water or food [38]. Several studies have been conducted on livestock and humans [39], but there is a lack of epidemiological data regarding wildlife [40]. In general, in raccoons, clinical presentation tends to be mild, consisting in intermittent diarrhea only in juveniles [33]. These data are consistent with the lack of typical intestinal lesions in the two adult raccoons found positive in the present study. The presence of cryptosporidiosis in raccoons poses a potential threat to public health, especially for people that manipulate and come into contact with these animals, considering the low host specificity of this parasite and its zoonotic relevance. 

*Eimeria* spp. infection in raccoons has been reported in several states in the USA (Illinois [41], Florida [20], and Ohio [42]) and in Japan [43]. In Europe, studies conducted in Germany by Winter et al. (2005) and Gey (1998) revealed different prevalence rates, ranging from 1.5% to 57.9% (for a review see [44]). Differently from *Cryptosporidium* spp., protozoa belonging to the genus *Eimeria* are non-zoonotic and characterized by a high host specificity, the most frequently isolated species in raccoons being *E. procyonis* and *E. nuttalli* [20]. To the best of our knowledge, this is the first report of *Cryptosporidium* sp. and *Eimeria* sp. infections in raccoons from Italy.

Among Nematodes, Ancylostomatidae were reported in raccoons by previous studies, most of them conducted in Asia, especially in Japan [45,46] and South Korea [47]. In Europe, the presence of these parasites has been reported in Poland [28,48] and Italy [3]. The isolated species were *Ancylostoma kusimaense*, *A. miyazakiense* [45,46], and *A. caninum*, in a case of co-infection with *Toxocara canis* [47]. Other studies did not report identification at a species level [20,27,48]. In our study, the investigation failed in recovering adult parasites. Although this family of parasites is widely reported in raccoons, there is no evidence on specific clinical signs or lesions in infected animals. These findings are consistent with the lack of lesions observed in the present study. 

Four identified species belonging to the Capillariidae family have been reported to date in raccoons: *C*. (*Pearsonema*) *plica* from urine samples [49,50,51,52], *C*. (*Eucoleus*) *aerophilus* from lungs [51], *C*. (*Aoncotheca*) *putorii* from the stomach [49,50,51], and *C. procyonis* from the esophageal mucosa [49,51,53] and the epithelial lining of the tongue [54,55]. In the present study, Capillariidae eggs evidenced in fecal samples through the Flotac^®^ technique were differentiated morphometrically from the genus *Trichuris,* based on the description of Di Cesare et al. in 2012 [56]. Capillariidae eggs were not identified at the species level; therefore, these could likely have been of respiratory (*E. aerophilus*) or gastrointestinal (*A. putorii*) origin; nevertheless, adult parasites were not retrieved in the gross or microscopic examination of these organ systems. Furthermore, no capillariid eggs or adults were retrieved during the nasal or pulmonary lavage fluids examination. Capillariid eggs were observed in the urinary sediment of six raccoons, but only a female adult parasite was retrieved in one out of six positive animals. Given that identification at the species level in the *Pearsonema* genus is based on the morphology of adult males [57], we refrained from classifying it as such. The prevalence of 9.6% observed in this study is in line with that reported in a recent study conducted on raccoons in Central Europe (3.7–8.7%) [52]. In Central Italy, *Pearsonema* sp. and particularly *P. plica* have been reported in several species sharing the same habitat of raccoons, such as red foxes (*Vulpes vulpes*), wolves (*Canis lupus*), pine martens (*Martes martes*), and European badgers (*Meles meles*) [24]. The proximity of raccoons to human settlements could pose the risk of urinary capillariosis infection for pet animals.

Regarding *B. procyonis*, in our study, one out of the three juvenile raccoons showing intestinal larvae did not reveal adult parasites in the ileal lumen, nor eggs shedding; this finding may suggest a prepatent stage of infection, as described by Kazacos in 2001 [2]. In raccoons infected by the ingestion of mature eggs, the infection evokes larval migration in the submucosa of the small intestine, and these larvae need up to 60 days before reentering in the intestinal lumen [2]. During this period, infected raccoons may not harbor adult parasites, nor shed eggs in the feces [2]. Our results highlight how the detection of first-stage larvae in the gut submucosa by histopathology can represent an important diagnostic tool in case of early-stage infections. All the *B. procyonis*-infected raccoons did not show evident gross lesions, except for the five cases of intestinal obstruction caused by the high burden of adult worms. At histopathology, apart from the detection of first-stage larvae, all the infected animals showed eosinophilic enteritis; these macroscopic and histopathological findings match with previous studies [2,18]. Chi-square tests did not show any significant relationship between the presence of *B. procyonis*, the parasite load, and the considered variables (sex, age, seasonality, and weight). Our findings only partially match with the previous literature, conducted mainly in North America. In endemic areas, *B. procyonis* usually show similar prevalence between male and female raccoons, but much higher prevalence in juvenile raccoons (> 90%) than in adults (40%–55%) [2]. Additionally, the average parasite burden is usually higher in young raccoons (mean 48–62, range 1–480) than adult raccoons (mean 12–22, range 1–257) [2]. This phenomenon is probably due to the life cycles of this parasite: young and juvenile raccoons are highly susceptible to egg infection, whereas adult raccoons become infected via intermediate host predation. Thus, in endemic areas, the parasite appears to be recruited into the raccoon population mainly through the orofecal route in young animals, which develop higher worm burdens and a higher prevalence of infection. Age resistance and increased intestinal local immunity may also contribute to the lower prevalence of *B. procyonis* in older raccoons. In addition, it is known that the parasite may undergo a yearly cycle in raccoons in temperate regions, with self-cure occurring in winter months, in which both prevalence and parasite burden strongly decrease. New infections usually rise in late spring and summer, and the overall prevalence peaks are reached in the fall. These seasonal changes are likely due to the dramatic reduction in food intake by raccoons in winter in northern temperate regions, leading to a strong reduction in their body weight and negatively impacting worm survival [2]. In our study, adult and young raccoons showed similar parasitic status and there were no significant seasonal differences; this phenomenon could be due to the low availability of infected paratenic hosts, so that both adult and young animals are exposed to the same orofecal route of infection; furthermore, the temperate and mild climatic changes among different seasons may attenuate seasonal variability. Extensive additional data and further investigations are required to better understand the epidemiology of *B. procyonis* in the Central Italy raccoon population.

In our study, from the QGIS analysis, we identified a core zone where the population of raccoons is seemingly concentrated. This area is characterized by the presence of mixed agricultural areas and woodlands, as well as several small residential areas with children’s playgrounds and parks. It is probable that the core zone is located around a putative site where the original animals escaped from captivity, in which the population has settled due to the abundance of alimentary resources. Considering that many species of paratenic hosts, both birds and mammals, including humans, can be infected by accidentally ingesting the eggs shed in the environment [2], this situation represents a public health risk. In paratenic hosts, the larvae can migrate to several tissues, including lungs and abdominal viscera (visceral larva migrans, VLM), eye (ocular larva migrans, OLM), and central nervous system (neural larva migrans, NLM) [2]. Raccoons (and occasionally canids) can also be infected by the ingestion of larvae by the predation or scavenging of paratenic hosts [2]. Moreover, raccoons are frequently found in close proximity to humans, making use of readily available habitat and food sources [58]; this behavior strongly increases the risk of human infection. The severity of human disease is influenced by the number of ingested eggs, ranging from severe and fatal neurologic syndromes to asymptomatic or paucisymptomatic outcomes [2]. It has to be considered that an infected raccoon can shed millions of eggs each day, as also reported in the present study (Table 1), which may remain infective for years, thus leading to a long-lasting contamination of the habitat [59].

In this scenario, the eradication of raccoons should be a priority, in particular in the identified core zone area. Working from a “one health” perspective, local authorities, following the first detection of this parasite, enforced an epidemiological surveillance program and implemented prevention measures. A risk communication campaign targeting different stakeholders in the area (hunters, veterinarians, wildlife technicians, and local health authorities) has been implemented. Operators were given detailed instructions concerning the proper handling of raccoons to minimize the risk of environmental contamination. The reporting of possible raccoon latrines, dead raccoons, and paratenic hosts by local residents and hikers in the area has been also encouraged. 

Finally, a serologic monitoring plan is also being developed and implemented for a preliminary screening and risk assessment of the exposed human population (veterinarians, wildlife and laboratory technicians, hunters, and people living nearby raccoon sightings).

## 5. Conclusions

The Central Italy non-native raccoon population has been found to carry multiple endoparasites, both Protozoa and Helminths. Most of these parasites exhibit a low host specificity, such as *Cryptosporidium* sp. or Capillaridae and Ancylostomatidae, therefore posing a risk of cross-infection with autochthonous wildlife and pets/livestock sharing the same habitat. In particular, the high prevalence of *B. procyonis* among the examined raccoon population represents a serious threat to public and animal health, due to the possible high environmental contamination, the complexity of its life cycle potentially involving different paratenic hosts, including humans and pets, and the possibility of severe or fatal clinical outcomes in infected human patients. 

The demonstrated role of the raccoon as a multi-parasite carrier should be an incentive to continuing the eradication/control of this alien species, and supports the need to implement related disease surveillance programs.

## Figures and Tables

**Table 1:** Distribution of the 62 raccoon carcasses in classes of body weight.

Body Weight Class (kg)	Number of Raccoons	%
0.0–1.9	5/62	8.06
2.0–3.9	15/62	24.19
4.0–5.9	24/62	38.70
6.0–7.9	15/62	24.19
≥8.0	4/62	6.45

**Table 2:** Quali-quantitative parasitological results from feces collected from the rectum and from urine sediments of the 62 raccoons.

Parasites	Technique	Prevalence	%	Eggs-Oocysts/g (m ± sd) *	Eggs-Oocysts/g (Min–Max)
*Eimeria* sp.	Flotac	2/62	3.2	4 ± 1.16	2–6
*Cryptosporidium* sp.	IF	2/62	3.2	-	-
*Baylisascaris procyonis*	Flotac	24/62	38.7	3852.30 ± 7355.30	2–28,320
Capillariidae	Flotac	6/62	9.6	4.7 ± 17.55	2–96
Ancylostomatidae	Flotac	2/62	3.2	2.5 ± 2.82	2–10
*Pearsonema* sp.	Urine centrifugation/NaCl flotation	6/62	9.6	-	-

* Confidence level 95%.

**Table 3:** Adult *B. procyonis* loads.

B. procyonis Load Range	Frequency (Number of Raccoons)	%
1–10	5/25	20
20–30	6/25	24
30–40	4/25	16
40–50	3/25	12
50–60	2/25	8
>100	5/25 *	20

* Raccoons with a load exceeding 100 adult parasites showed intestinal obstruction.

**Table 4:** Number of trapped raccoons in each trapping site.

Number of Captured Raccoons Per Trap	Number of Traps	Total Number of Captured Raccoons
1	22	22
2	6	12
3	1	3
4	2	8
15	1	15
**Total**	**32**	**60**

**Table 5:** Statistical analysis showing no relationship between *B. procyonis* positivity and the four considered variables (sex, age, seasonality, weight).

Variables	Test	*p*
Sex	Χ^2^ (1, N = 62) = 0.47683	*p* = 0.4899
Age	Χ^2^ (1, N = 62) = 0.01242	*p* = 0.9113
Seasonality	Χ^2^ (3, N = 62) = 2.9039	*p* = 0.4067
Weight	Χ^2^ (3, N = 62) = 1.2647	*p* = 0.7375

## Data Availability

One of the sequences obtained in this study is publicly available on the European Nucleotide167 Archive (ENA) under the accession number ERZ4009650. All other data generated or analyzed during this study are included in this published article.

## Supplementary Materials

The following supporting information can be downloaded at: https://www.mdpi.com/article/10.3390/vetsci10020171/s1, Figure S1: Histological cross sections of larvae of *B. procyonis* in the intestinal wall of a raccoon; Figure S2: Histological section of raccoon intestine. Larva of *B. procyonis* (L) surrounded by inflammatory exudate consisting of macrophages (black arrowheads), lymphocytes (asterisks), and cellular debris. Table S1: Positive and negative raccoons with their respective trapping sites.
