# Supplementary material for: Detection of Endoparasites in Non-Native Raccoons from Central Italy
Source: Vet Sci. 2023 Feb 20;10(2):171. doi: 10.3390/vetsci10020171 (PMC9961332; doi:10.3390/vetsci10020171)
Supplement: Supplementary file 1 [file vetsci-10-00171-s001.zip › Supplementary material/Supplementary material Table S1.pdf]

Table S1. Positive and negative raccoons with their respective trapping site.

Trap ID	Raccoons		
	Negative	Positive	Total
1	0	1	1
2	1	0	1
3	1	0	1
4	1	1	2
5	1	1	2
6	0	1	1
7	1	1	2
8	0	1	1
9	1	0	1
10	1	0	1
11	1	0	1
12	1	0	1
13	1	0	1
14	1	0	1
15	1	0	1
16	1	0	1
17	1	0	1
18	0	4	4
19	1	14	15
20	1	1	2
21	1	0	1
22	3	0	3
23	2	0	2
24	1	0	1
25	0	1	1
26	1	0	1
27	2	0	2
28	1	0	1
29	4	0	4
30	1	0	1
31	1	0	1
32	1	0	1
**Total**	**34**	**26**	**60**
