# Supplementary figures and images for: Detection of Endoparasites in Non-Native Raccoons from Central Italy
Source: Vet Sci. 2023 Feb 20;10(2):171. doi: 10.3390/vetsci10020171 (PMC9961332; doi:10.3390/vetsci10020171)

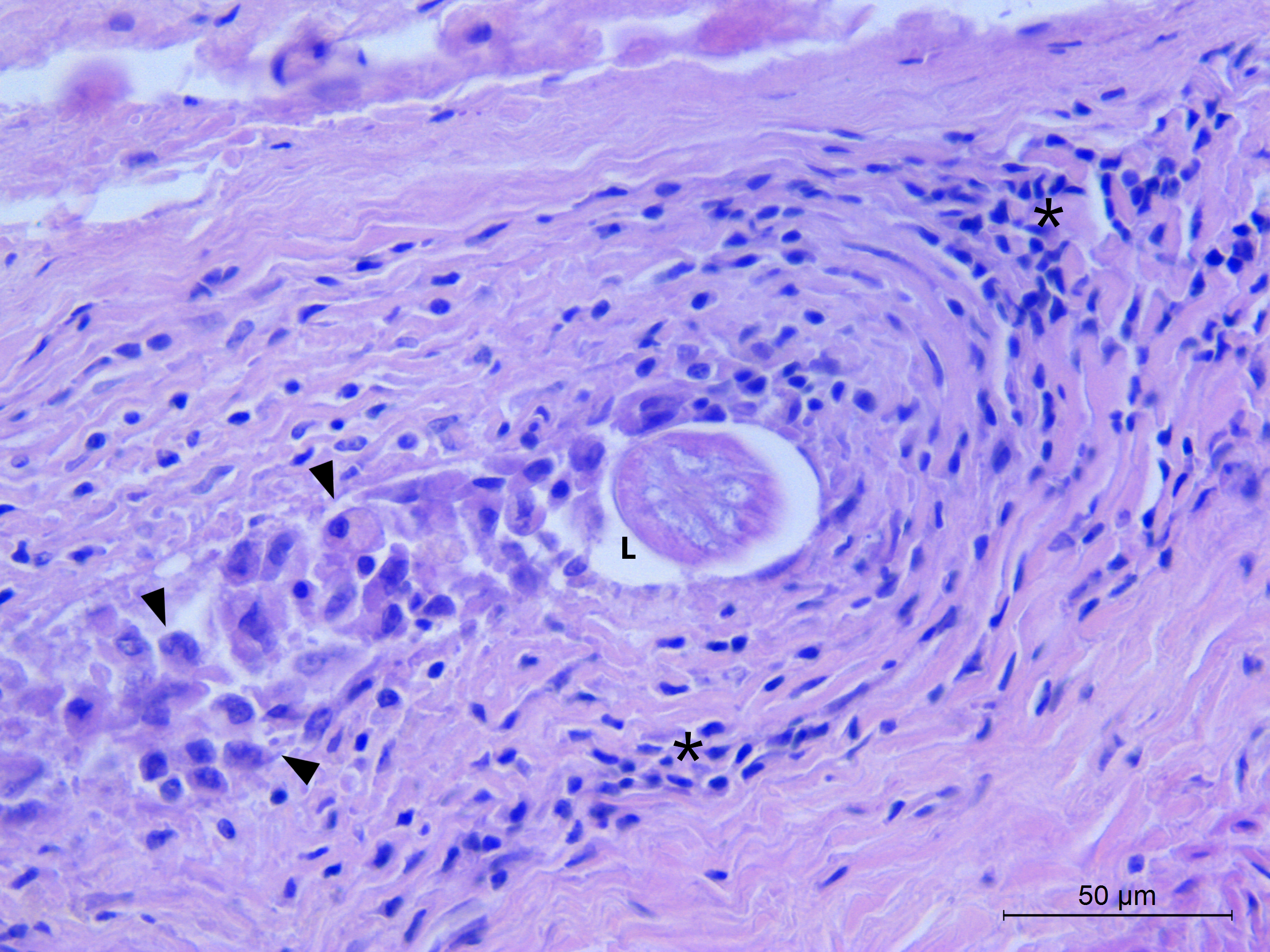

Supplement: Supplementary file 1 [file vetsci-10-00171-s001.zip › Supplementary material/S2 .tiff]

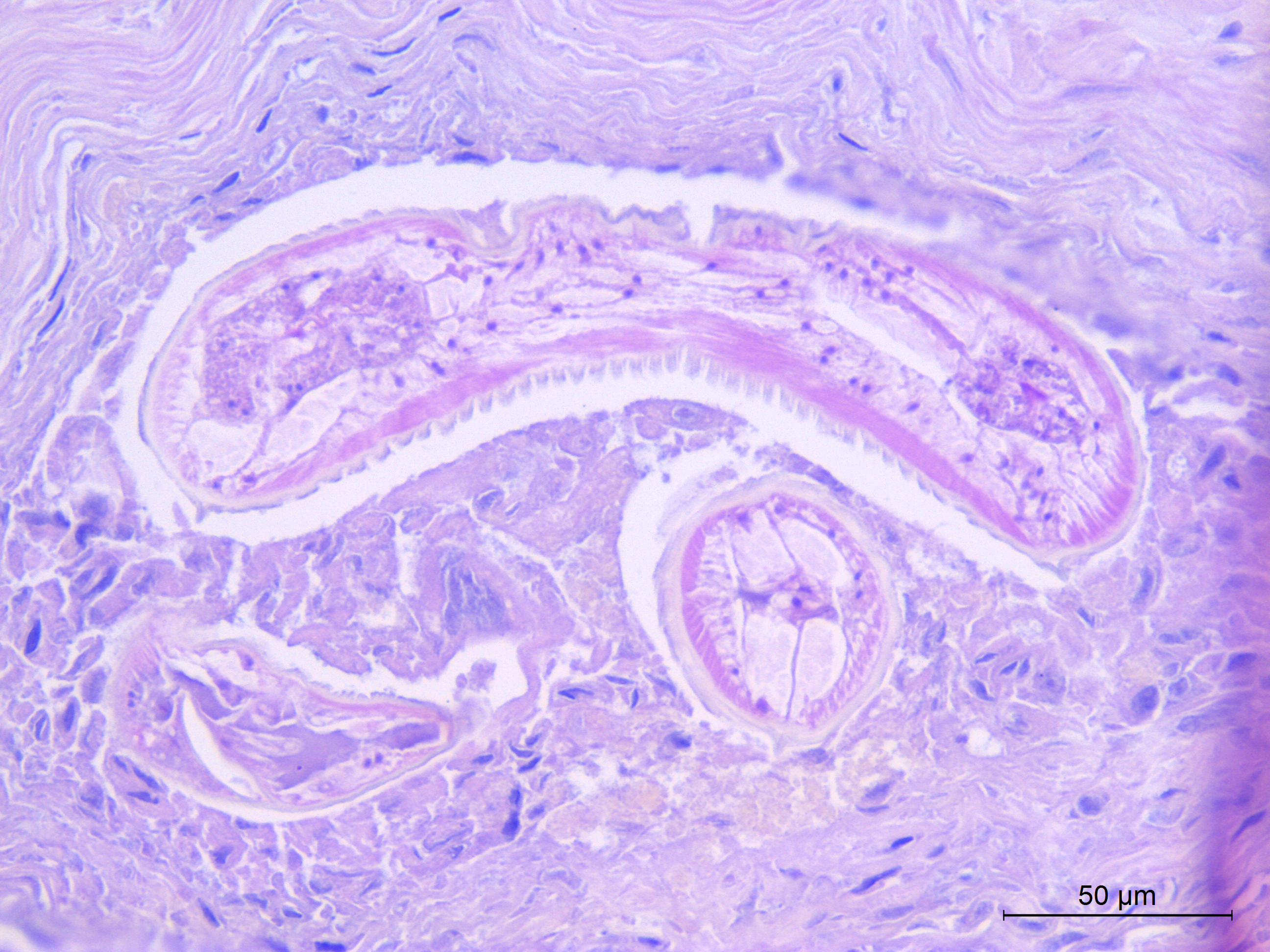

Supplement: Supplementary file 1 [file vetsci-10-00171-s001.zip › Supplementary material/S1.tif]
